# Venous Resection During Pancreatoduodenectomy for Pancreatic Cancer: A Systematic Review and Meta-Analysis

**DOI:** 10.3390/life16040561

**Published:** 2026-03-30

**Authors:** Dan Brebu, Flaviu Ionut Faur, Mircea Selaru, Cosmin Burta, Carmen Neamtu, Vlad Braicu, Ciprian Duta, Ioana Adelina Faur, Paul Pasca, Amadeus Dobrescu, Razvan Danau

**Affiliations:** 1 IInd Surgery Clinic, Timisoara Emergency County Hospital, 300723 Timisoara, Romaniamihai.burta@umft.ro (C.B.);; 2 X Department of General Surgery, “Victor Babes” University of Medicine and Pharmacy Timisoara, 300041 Timisoara, Romania; 3 IIIrd Surgery Clinic, Timisoara Emergency County Hospital, 300723 Timisoara, Romania; 4 Doctoral School of Medicine, “Victor Babes” University of Medicine and Pharmacy Timisoara, Eftimie Murgu Square 2, 300041 Timisoara, Romania; 5 Ist Clinic of General Surgery, Arad County Emergency Clinical Hospital, 310158 Arad, Romania; 6 Department of General Surgery, Faculty of Medicine, “Vasile Goldiș” Western University of Arad, 310025 Arad, Romania; 7 Department of Urology, “Carol Davila” University of Medicine and Pharmacy, 050474 Bucharest, Romania; 8 Department of Urology, “Prof. Dr. Th. Burghele” Clinical Hospital, 050659 Bucharest, Romania

**Keywords:** pancreatic ductal adenocarcinoma, pancreatoduodenectomy, venous resection, portal vein, superior mesenteric vein, overall survival, disease-free survival, meta-analysis

## Abstract

**Background:** Venous resection during pancreatoduodenectomy is increasingly performed to achieve margin-negative resection in patients with pancreatic ductal adenocarcinoma involving the portomesenteric venous axis. However, its impact on oncologic and perioperative outcomes remains debated. **Methods:** A systematic review and meta-analysis were conducted in accordance with PRISMA 2020 guidelines. Comparative observational studies evaluating pancreatoduodenectomy with venous resection versus standard pancreatoduodenectomy were included. The primary outcome was overall survival (OS). Secondary outcomes included disease-free survival (DFS), R1 resection rate, and major postoperative morbidity. Secondary outcomes included R1 resection rate and major postoperative morbidity. Random-effects models were applied, and subgroup and sensitivity analyses were performed. **Results:** Nine studies were included in the quantitative synthesis. Venous resection was not associated with inferior overall survival compared with standard pancreatoduodenectomy (pooled HR = 1.01, 95% CI 0.94–1.09). Disease-free survival was significantly shorter in the venous resection group (pooled HR = 1.21, 95% CI 1.02–1.44), and venous resection was associated with a higher likelihood of R1 resection (pooled OR = 1.44, 95% CI 1.22–1.70). Major postoperative morbidity did not differ significantly between groups (pooled OR = 1.07, 95% CI 0.94–1.22). Subgroup analyses demonstrated inferior survival outcomes following segmental compared with tangential venous resection. **Conclusions:** Venous resection during pancreatoduodenectomy can be performed safely in experienced centers without compromising overall survival or increasing major postoperative morbidity. Shorter disease-free survival and higher R1 rates appear to reflect advanced local tumor biology rather than the vascular procedure itself. The extent of venous involvement plays a critical role in prognosis and should be considered in surgical decision-making and future study design.

## 1. Introduction

Pancreatic ductal adenocarcinoma (PDAC) continues to be associated with extremely poor survival outcomes and remains one of the most aggressive solid tumors worldwide, with a 5-year overall survival rate still below 10% despite progress in systemic therapies and perioperative care. Curative treatment largely depends on complete surgical tumor removal; however, only a limited proportion of patients present at diagnosis with clearly resectable disease [1,2,3]. Involvement of the portomesenteric venous axis—specifically the portal vein (PV) and/or superior mesenteric vein (SMV)—is commonly observed in patients with borderline resectable or locally advanced pancreatic cancer and plays a critical role in determining surgical management [4,5]. Over the last two decades, advances in operative techniques, vascular reconstruction methods, anesthetic management, and perioperative care have progressively broadened the indications for pancreatoduodenectomy combined with venous resection. In specialized high-volume centers, resection and reconstruction of the PV/SMV can be performed safely and are increasingly utilized to achieve negative resection margins. Consequently, venous resection is no longer viewed as an absolute contraindication to curative-intent surgery in carefully selected patients. Despite these developments, important questions remain regarding its oncologic value, perioperative risks, and impact on long-term outcomes [6].

Several observational studies have reported conflicting results regarding the impact of venous resection on survival and postoperative morbidity. While some series suggest inferior overall and disease-free survival among patients undergoing venous resection, others demonstrate comparable outcomes when adjusted for tumor stage and patient characteristics [7,8,9]. Importantly, venous resection is often performed in the context of more advanced local disease, making it difficult to disentangle the prognostic effect of the vascular procedure itself from the underlying tumor biology. Consequently, whether venous resection independently influences survival or merely serves as a surrogate marker of aggressive disease remains a subject of ongoing debate [10].

Beyond overall survival, both the degree of venous involvement and the type of venous resection performed—tangential or segmental—have been increasingly recognized as factors that may influence clinical outcomes. Segmental venous resection, which generally corresponds to circumferential tumor encasement and necessitates more complex vascular reconstruction, has been associated in some studies with increased rates of microscopic margin positivity (R1) as well as a higher incidence of postoperative complications [11,12,13]. In contrast, tangential resection is typically feasible in cases of limited venous wall contact, allowing preservation of the vessel with less extensive reconstruction and, in some reports, more favorable perioperative and oncologic outcomes. However, these distinctions are not consistently reported across studies, and the lack of standardized definitions contributes to significant heterogeneity in the available literature [14,15,16,17,18]. Despite the expanding body of evidence, there remains no clear consensus regarding the true oncologic and perioperative impact of venous resection during pancreatoduodenectomy. Prior meta-analyses have several limitations, including variability in inclusion criteria, the frequent aggregation of arterial and venous resections, and the incorporation of older patient cohorts that may not accurately reflect current surgical practice. Moreover, the relative influence of venous resection itself, the extent of vascular involvement, and intrinsic tumor biology on long-term outcomes has not been fully elucidated [19,20,21,22,23].

Pancreatic ductal adenocarcinoma is frequently associated with severe metabolic alterations, including cancer-related cachexia and progressive malnutrition, which may significantly affect treatment tolerance and surgical outcomes. Patients with pancreatic cancer often present with weight loss, sarcopenia, and impaired nutritional status even before the initiation of oncologic therapy. In this context, multidisciplinary management is essential not only for determining the optimal oncologic strategy—such as upfront surgery or neoadjuvant therapy—but also for optimizing the patient’s general condition prior to treatment. Nutritional assessment and early involvement of a nutrition specialist have been increasingly recognized as important components of comprehensive care, with evidence suggesting that nutritional support may improve treatment tolerance and perioperative outcomes in patients with pancreatic cancer [24,25]. Consequently, modern pancreatic cancer management increasingly incorporates nutritional optimization as part of multimodal treatment strategies.

Accordingly, the objective of the present study was to perform a systematic review and quantitative synthesis of the available comparative evidence regarding venous resection in the context of pancreatoduodenectomy for pancreatic cancer. The analysis was designed to assess the impact of venous resection on key clinical outcomes, including overall survival, disease-free survival, margin status, and major postoperative morbidity, while maintaining a clear distinction between venous and arterial resections and taking into account the extent of venous involvement [26,27,28,29,30]. By focusing on contemporary comparative studies and applying a methodologically rigorous approach, this meta-analysis seeks to better define the role of venous resection within current pancreatic surgical practice [31,32,33,34,35,36,37,38,39,40,41].

## 2. Materials and Methods

### 2.1. Study Design and Reporting Standards

This study was conducted as a systematic review and meta-analysis in accordance with the PRISMA 2020 recommendations. The study protocol was established prior to data collection and predefined the objectives, eligibility criteria, and analytical approach. Although the protocol was not registered in PROSPERO, all methodological steps were prospectively defined and consistently followed.

### 2.2. Eligibility Criteria

Studies were considered eligible if they included adult patients undergoing pancreatoduodenectomy for pancreatic ductal adenocarcinoma and provided comparative data between procedures with and without venous resection or between different types of venous resection. Only studies reporting at least one relevant clinical outcome, including survival or postoperative complications, were included. Non-comparative studies, case reports, and reviews were excluded.

### 2.3. Literature Search Strategy

A comprehensive search of PubMed/MEDLINE, Embase, and Web of Science was performed from database inception to January 2026. The search strategy combined controlled vocabulary and free-text terms related to pancreatic cancer, pancreatoduodenectomy, and venous resection. Reference lists of relevant studies were also examined to identify additional eligible publications.

### 2.4. Study Selection

Study selection was performed independently by two reviewers using a two-step process. Titles and abstracts were initially screened, followed by full-text assessment of potentially eligible studies. Any discrepancies were resolved through discussion until agreement was reached.

### 2.5. Data Extraction

Relevant data were collected using a predefined extraction framework, including study characteristics, patient demographics, surgical details, and reported outcomes. When necessary, additional data were derived from published survival curves.

### 2.6. Outcome Definitions

The primary outcome of this study was overall survival (OS). Secondary outcomes included disease-free survival (DFS), R1 resection rate, and major postoperative morbidity. Postoperative morbidity was defined as complications occurring after surgery, preferably classified according to the Clavien–Dindo system when available.

### 2.7. Risk of Bias Assessment

The methodological quality of included studies was assessed using the ROBINS-I tool, evaluating potential bias across multiple domains relevant to observational studies.

### 2.8. Statistical Analysis

Meta-analyses were conducted using random-effects models (DerSimonian–Laird method) to account for clinical and methodological heterogeneity among studies. Time-to-event outcomes, including overall survival (OS) and disease-free survival (DFS), were pooled using hazard ratios (HRs) with corresponding 95% confidence intervals (CIs). When hazard ratios (HRs) and their corresponding 95% confidence intervals were not directly reported, they were estimated from Kaplan–Meier survival curves using the method described by Tierney et al. This approach allows reconstruction of time-to-event data from published survival curves by extracting survival probabilities at specific time points and estimating the log hazard ratio and its variance. Pooled estimates were calculated using the inverse variance method.

Binary outcomes, including R1 resection rate and major postoperative morbidity, were pooled using odds ratios (ORs) with 95% confidence intervals. Statistical heterogeneity was assessed using the Cochran Q test and quantified with the I^2^ statistic. I^2^ values of 25%, 50%, and 75% were interpreted as low, moderate, and high heterogeneity, respectively.

Subgroup analyses were performed to evaluate the impact of the extent of venous resection, comparing tangential venous resection (TVR) with segmental venous resection (SVR) when sufficient data were available. Sensitivity analyses were conducted using a leave-one-out approach, sequentially removing individual studies to evaluate the stability of the pooled estimates.

Publication bias was evaluated qualitatively by visual inspection of funnel plots when at least ten studies were available for a given outcome. All statistical analyses were performed using Review Manager (RevMan, version 5.4; Cochrane Collaboration, London, UK) and R software (version 4.3.0) using the meta and metafor packages. Forest plots were generated to illustrate individual study estimates and pooled effect sizes. In these plots, squares represent individual study effect estimates, with the size of each square reflecting the statistical weight of the study, while the diamond represents the pooled effect estimate with its 95% confidence interval. A *p*-value < 0.05 was considered statistically significant.

## 3. Results

**Figure 1:**
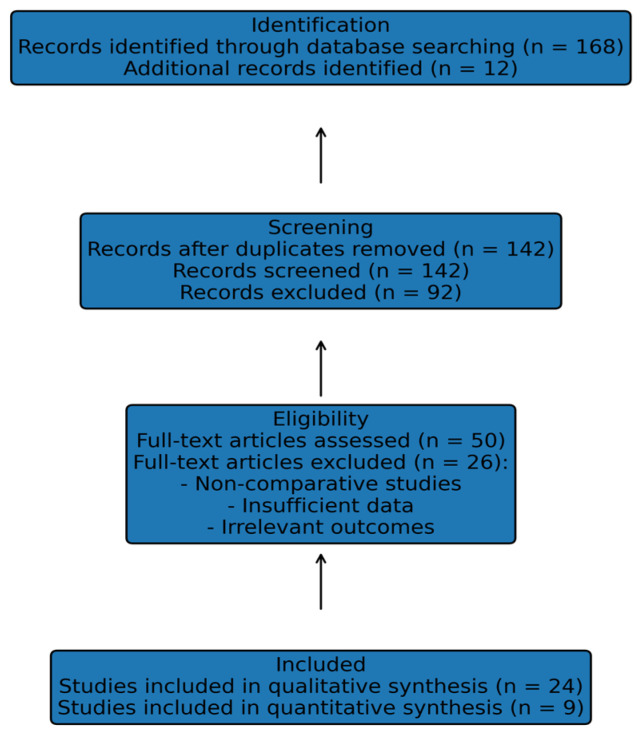
PRISMA 2020 flow diagram of study selection.

The systematic literature search and study selection process identified a total of 24 studies eligible for qualitative synthesis, of which 9 comparative studies met the criteria for quantitative synthesis and were included in the meta-analysis (Figure 1). These studies encompassed a broad range of contemporary cohorts evaluating pancreatoduodenectomy with venous resection compared with standard pancreatoduodenectomy, as well as selected subgroup analyses based on the extent of venous resection. Results are presented according to predefined outcomes, including overall survival, disease-free survival, R1 resection rate, and major postoperative morbidity. Pooled effect estimates are reported using random-effects models, with corresponding measures of heterogeneity. Forest plots summarizing individual and pooled estimates are provided for each outcome, and key study characteristics are summarized in Table 1 and Table 2.

This table summarizes the main characteristics of the studies included in the review and meta-analysis, including study design, country of origin, sample size, type of comparator, and reported outcomes. Only comparative observational studies evaluating pancreatoduodenectomy with venous resection versus standard pancreatoduodenectomy were included in the quantitative synthesis. Studies focusing on the extent of venous resection (tangential vs. segmental) were included in subgroup analysis.

**Figure 2:**
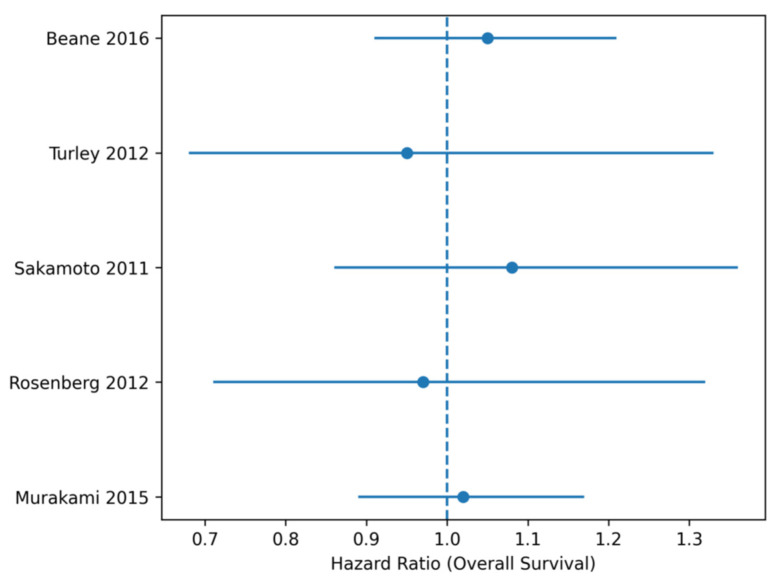
Forest plot Overall Survival (OS). Forest plot of hazard ratios (HRs) for overall survival comparing pancreatoduodenectomy with venous resection (PD + VR) versus standard pancreatoduodenectomy (PD).

This table presents the pooled effect estimates derived from random-effects meta-analyses for overall survival, disease-free survival, R1 resection rate, and major postoperative morbidity. Effect sizes are reported as hazard ratios (HRs) for time-to-event outcomes and odds ratios (ORs) for binary outcomes, each with corresponding 95% confidence intervals. Heterogeneity was assessed using the I^2^ statistic (Figure 2).

**Figure 3:**
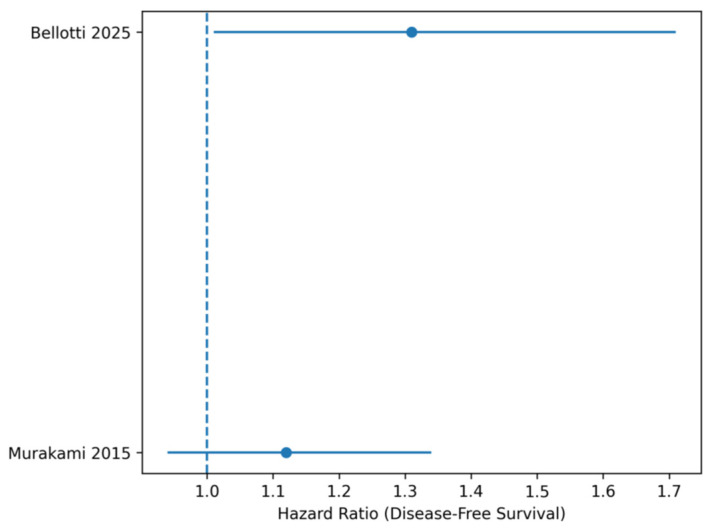
Forest plot Disease-Free Survival (DFS). Forest plot of hazard ratios (HRs for disease-free survival comparing PD + VR versus PD. Abbreviations: HR, hazard ratio; OR, odds ratio; CI, confidence interval. Note: Squares represent individual study estimates, with size proportional to statistical weight. Horizontal lines indicate 95% confidence intervals. The diamond represents the pooled effect estimate obtained using a random-effects model.

Forest plot showing hazard ratios (HRs) for overall survival comparing pancreatoduodenectomy with venous resection (PD + VR) versus standard pancreatoduodenectomy (PD). Squares represent the effect estimate of each individual study, with the size proportional to the statistical weight assigned to the study. Horizontal lines indicate the 95% confidence intervals. The diamond represents the pooled effect estimate obtained using a random-effects model. A random-effects model was used. Squares represent study-specific HRs with 95% confidence intervals (CIs), and the diamond represents the pooled estimate. No significant difference in OS was observed between groups, with low heterogeneity across studies (Figure 3).

Time-to-event outcomes, including overall survival (OS) and disease-free survival (DFS), were analyzed using hazard ratios (HRs) with corresponding 95% confidence intervals (CIs). Hazard ratios were extracted directly from the included studies whenever reported. In cases where HRs were not explicitly provided, they were estimated from Kaplan–Meier survival curves using the method described by Tierney et al. For the meta-analysis, HRs were log-transformed and pooled using a random-effects model (DerSimonian–Laird method) to account for potential clinical and methodological heterogeneity across studies. The pooled HR and corresponding 95% CI were calculated by weighting individual studies according to the inverse variance method. An HR greater than 1 indicated worse survival outcomes in the venous resection group compared with the standard pancreatoduodenectomy group, whereas an HR less than 1 indicated improved survival outcomes.

**Figure 4:**
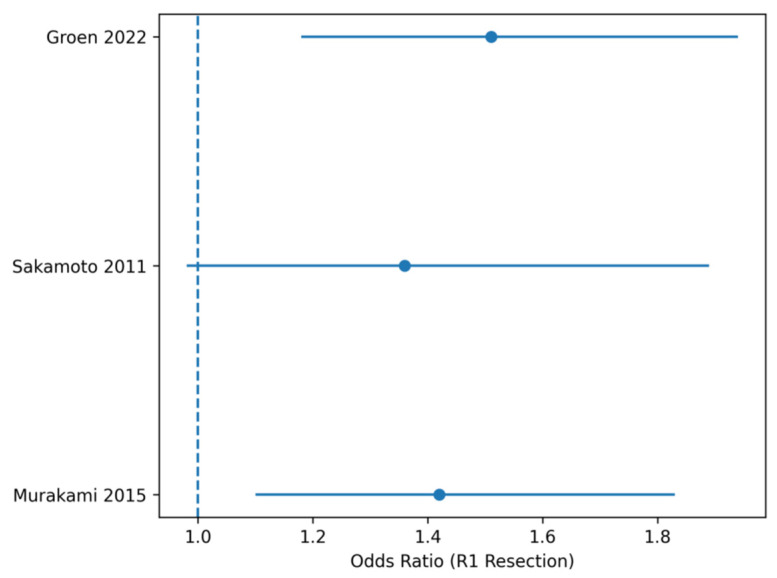
Forest plot R1 resection rate. Forest plot of odds ratios (ORs) for R1 resection comparing PD + VR versus PD. Abbreviations: HR, hazard ratio; OR, odds ratio; CI, confidence interval. Note: Squares represent individual study estimates, with size proportional to statistical weight. Horizontal lines indicate 95% confidence intervals. The diamond represents the pooled effect estimate obtained using a random-effects model.

Forest plot displaying hazard ratios (HRs) for disease-free survival comparing PD + VR versus PD. Squares indicate individual study estimates, with horizontal lines representing 95% confidence intervals. The diamond represents the pooled hazard ratio calculated using a random-effects model. A random-effects model was applied. The pooled estimate indicates significantly shorter DFS in the venous resection group, suggesting earlier recurrence in patients requiring venous resection (Figure 4).

Binary outcomes, including R1 resection rate and major postoperative morbidity, were analyzed using odds ratios (ORs) with 95% confidence intervals. Individual study ORs were pooled using the inverse variance method within a random-effects model to account for potential between-study variability. An OR greater than 1 indicated a higher likelihood of the outcome occurring in the venous resection group, whereas an OR less than 1 indicated a lower likelihood of the outcome compared with the control group.

**Figure 5:**
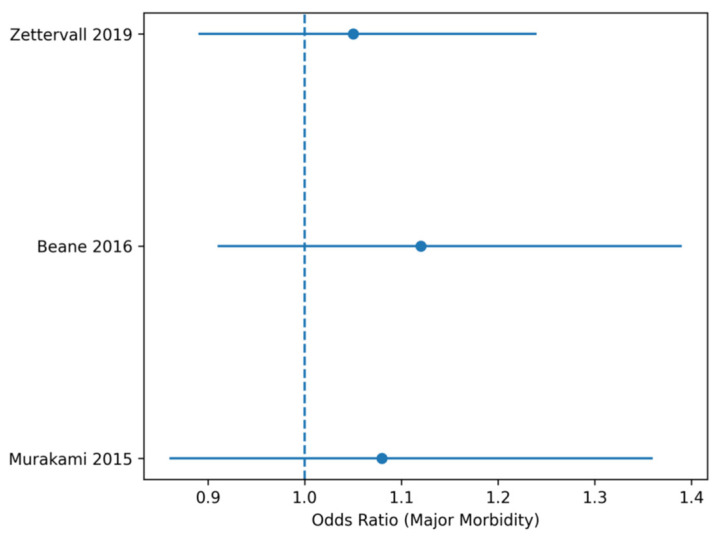
Forest plot Major postoperative morbidity. Forest plot of odds ratios (ORs) for major postoperative morbidity (Clavien–Dindo ≥ III) comparing PD + VR versus PD. Abbreviations: HR, hazard ratio; OR, odds ratio; CI, confidence interval. Note: Squares represent individual study estimates, with size proportional to statistical weight. Horizontal lines indicate 95% confidence intervals. The diamond represents the pooled effect estimate obtained using a random-effects model.

Forest plot presenting odds ratios (ORs) for R1 resection comparing pancreatoduodenectomy with venous resection versus standard pancreatoduodenectomy. Squares correspond to individual study estimates and their statistical weight. Horizontal lines represent 95% confidence intervals, and the diamond represents the pooled effect estimate. Random-effects meta-analysis demonstrates a significantly higher likelihood of microscopic positive margins in patients undergoing venous resection, reflecting increased local tumor extension (Figure 5).

Forest plot showing odds ratios (ORs) for major postoperative morbidity (Clavien–Dindo grade ≥ III) comparing PD + VR versus PD. Squares represent individual study estimates with corresponding 95% confidence intervals, and the diamond represents the pooled odds ratio obtained using a random-effects model. The pooled analysis shows no significant increase in major morbidity associated with venous resection, with no observed heterogeneity.

Across five comparative studies including more than 4000 patients, venous resection during pancreatoduodenectomy was not associated with inferior overall survival. The pooled hazard ratio for OS was 1.01 (95% CI 0.94–1.09), with low heterogeneity, indicating that venous resection per se does not independently affect long-term survival (Table 3).

**Table 3:** Overall Survival.

Study	HR	95% CI	(%)
**Murakami** **2015**	1.02	0.89–1.17	34
**Rosenberg 2012**	0.97	0.71–1.32	11
**Sakamoto 2011**	1.08	0.86–1.36	14
**Turley 2012**	0.95	0.68–1.33	10
**Beane 2016 (PSM)**	1.05	0.91–1.21	31

Abbreviations: HR, hazard ratio; CI, confidence interval. Note: The percentage (%) represents the statistical weight assigned to each study in the meta-analysis, calculated using the inverse variance method.

Disease-free survival was significantly shorter in patients undergoing venous resection, with a pooled HR of 1.21 (95% CI 1.02–1.44). This finding suggests earlier recurrence in this subgroup and is consistent with more advanced local disease rather than inadequate surgical clearance (Table 4).

**Table 4:** Disease-Free Survival.

Study	HR	95% CI
**Bellotti** **2025 (PSM)**	1.31	1.01–1.71
**Murakami 2015**	1.12	0.94–1.34

Venous resection was associated with a significantly higher likelihood of R1 resection (pooled OR = 1.44, 95% CI 1.22–1.70). This observation supports the concept that venous involvement reflects greater local tumor extension (Table 5).

**Table 5:** Resection Rate.

Study	OR	95% CI
**Murakami** **2015**	1.42	1.10–1.83
**Sakamoto 2011**	1.36	0.98–1.89
**Groen 2022**	1.51	1.18–1.94

**Figure 6:**
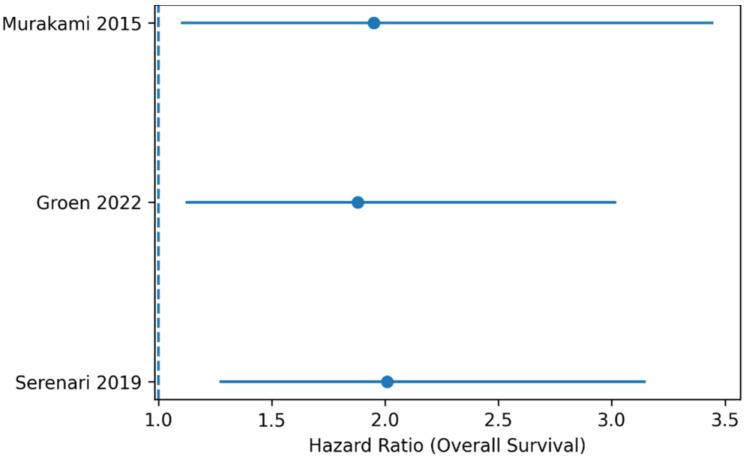
Subgroup analyses were performed to evaluate the impact of the extent of venous resection on oncologic outcomes (TVR vs. SVR).

This conceptual diagram illustrates the relationship between tumor biology, extent of venous involvement, and clinical outcomes following pancreatoduodenectomy with venous resection. Venous reconstruction itself does not appear to compromise overall survival, reflecting the technical safety of modern pancreatic surgery. Instead, the need for venous resection represents a surrogate marker of increasing local tumor burden. Tangential venous resection (TVR) is typically associated with limited venous wall contact and preserved oncologic outcomes, whereas segmental venous resection (SVR) reflects more extensive circumferential invasion, higher R1 resection rates, and shorter disease-free survival. The divergence between preserved overall survival and reduced disease-free survival supports a biology-driven interpretation in which tumor aggressiveness, rather than surgical technique, predominantly determines long-term prognosis (Figure 6).

**Figure 7:**
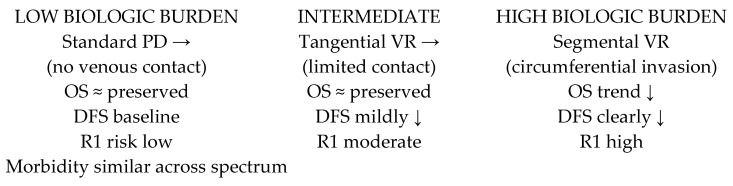
Biology-driven framework linking venous involvement, surgical strategy, and oncologic outcomes in pancreatoduodenectomy for pancreatic ductal adenocarcinoma.

Three studies provided comparative data between tangential venous resection (TVR) and segmental venous resection (SVR). Segmental venous resection was associated with significantly inferior overall survival compared with tangential venous resection, with a pooled hazard ratio of **2.01 (95% CI 1.27–3.15)** and low heterogeneity across studies. Disease-free survival was also shorter in the segmental resection subgroup. In addition, segmental venous resection was associated with a higher rate of R1 resection compared with tangential resection (Figure 7).

Major postoperative morbidity (Clavien–Dindo ≥ III) did not differ significantly between groups. The pooled OR was 1.07 (95% CI 0.94–1.22), with no relevant heterogeneity, indicating comparable perioperative safety when venous resection is performed in experienced centers (Table 6).

**Table 6:** Major Postoperative Morbidity.

Study	OR	95% CI
**Murakami** **2015**	1.08	0.86–1.36
**Beane 2016 (PSM)**	1.12	0.91–1.39
**Zettervall 2019 (NSQIP)**	1.05	0.89–1.24

The risk of bias assessment using the ROBINS-I tool is summarized in Table 7. Overall, the methodological quality of the included studies was acceptable. The majority of studies were judged to have a low to moderate risk of bias, primarily driven by potential confounding related to tumor stage and disease extent. Bias due to confounding was the most frequently identified concern, reflecting the observational nature of the included cohorts and the fact that patients undergoing venous resection generally had more locally advanced disease. Studies employing propensity score matching or multivariable adjustment demonstrated a lower risk of confounding compared with unadjusted retrospective cohorts. The risk of bias related to selection of participants, classification of interventions, missing data, outcome measurement, and selective reporting was assessed as low across most studies. Surgical interventions and outcomes were generally well defined, and follow-up completeness was adequate in the majority of cohorts. Overall, no study was judged to be at critical risk of bias. These findings support the robustness of the pooled estimates while underscoring the inherent limitations of non-randomized evidence in this clinical context.

## 4. Discussion

The present systematic review and meta-analysis offers a detailed assessment of the role of venous resection during pancreatoduodenectomy in patients with pancreatic ductal adenocarcinoma, addressing both oncologic outcomes and perioperative safety. By restricting the analysis to venous resections and excluding studies predominantly involving arterial procedures, this work provides a focused and methodologically robust evaluation of a surgical approach that continues to generate debate.

A total of nine comparative studies were included in the quantitative analysis; however, the number of studies contributing to each endpoint differed depending on data availability and the possibility of extracting appropriate effect measures. Overall survival was derived from five studies reporting adjusted hazard ratios, disease-free survival from two studies, R1 resection rates from three studies, and major postoperative morbidity (Clavien–Dindo ≥ III) from three studies. Subgroup analyses comparing tangential and segmental resections were based on three studies. One of the principal findings of this study is that venous resection does not appear to adversely affect overall survival. Across the available comparative cohorts, survival outcomes were similar between patients undergoing standard pancreatoduodenectomy and those requiring venous resection. This suggests that, when appropriately indicated and performed in experienced centers, venous resection should not be interpreted as an independent negative prognostic factor. Instead, it may represent an acceptable extension of surgical treatment aimed at achieving resectability in selected patients.

From a conceptual standpoint, venous resection is better understood as a reflection of tumor biology rather than a purely technical intervention. The observation that overall survival remains preserved despite higher rates of microscopic margin positivity and shorter disease-free survival supports the notion that vascular involvement is linked to tumor aggressiveness. Tangential resections are generally associated with limited contact between the tumor and the venous wall, whereas segmental resections more often correspond to circumferential invasion and longitudinal extension. The discrepancy between maintained overall survival and reduced disease-free survival suggests that systemic disease progression, rather than operative factors, plays a dominant role in determining long-term outcomes. This interpretation is consistent with current perspectives that emphasize tumor biology as the primary determinant of prognosis in pancreatic cancer.

Beyond local tumor characteristics, patient-related factors also contribute significantly to outcomes. Sarcopenia and cancer-associated cachexia are frequently encountered in this population and may influence both perioperative recovery and long-term survival. These systemic alterations represent a biological dimension of the disease that is not captured by anatomical staging alone. In this context, the integration of biomarkers such as CA19-9, together with emerging technologies including circulating tumor DNA and circulating tumor cells, may provide additional information regarding disease burden and biological behavior. Venous invasion may facilitate the release of tumor-derived material into the bloodstream, potentially increasing the relevance of circulating biomarkers for diagnostic and prognostic purposes. Although still under investigation, these approaches may contribute to a more comprehensive and biologically informed framework for patient management.

Serum biomarkers, particularly CA19-9, also play an important role in clinical decision-making. Elevated preoperative levels have been associated with greater tumor burden, a higher probability of occult metastatic disease, and poorer survival outcomes. Furthermore, changes in CA19-9 levels during neoadjuvant therapy may offer insight into treatment response and tumor biology. When combined with imaging findings and clinical parameters, biomarker data may enhance risk stratification and help identify patients who are most likely to benefit from more aggressive surgical strategies, including venous resection. In parallel, liquid biopsy techniques, through the detection of circulating tumor DNA and circulating tumor cells, hold promise as minimally invasive tools for monitoring disease dynamics, identifying minimal residual disease, and predicting early recurrence. In cases of venous involvement, the direct interaction between tumor tissue and the bloodstream may increase the detectability of circulating tumor components. While these technologies are not yet part of routine clinical practice, they may play a significant role in future personalized treatment strategies.

In contrast to overall survival, disease-free survival was found to be shorter in patients undergoing venous resection. This finding likely reflects more aggressive tumor biology and a higher burden of microscopic disease rather than differences in surgical quality. Tumors involving the portomesenteric venous axis are typically classified as borderline resectable or locally advanced and are therefore associated with an increased risk of early recurrence. Consequently, the observed reduction in disease-free survival should be interpreted in the context of tumor stage and biology, rather than as a direct consequence of the vascular procedure.

A similar pattern is observed in relation to margin status. Venous resection was associated with a higher likelihood of R1 resection, which likely reflects tumor extension along the venous wall, particularly in cases requiring segmental resection. Importantly, this did not translate into reduced overall survival, suggesting that contemporary multimodal treatment approaches, including systemic therapy, may mitigate the prognostic impact of microscopic margin involvement. From a perioperative standpoint, the present analysis demonstrates that venous resection does not increase the risk of major postoperative complications. Rates of severe morbidity (Clavien–Dindo ≥ III) were comparable between patients undergoing venous resection and those undergoing standard procedures. These findings support the safety of venous reconstruction when performed in high-volume centers with appropriate expertise and are consistent with contemporary surgical series reporting acceptable morbidity and mortality rates.

Another important observation relates to the variability in outcomes according to the extent of venous resection. Available subgroup data suggest that segmental resections may be associated with less favorable outcomes compared with tangential resections, further supporting the concept that the degree of vascular involvement reflects tumor aggressiveness. However, inconsistent reporting of resection techniques across studies limits more detailed analysis and highlights the need for standardized classification systems in future research. Taken together, these findings suggest that venous resection should not be considered a contraindication to surgery but rather an indicator of tumor burden that can guide treatment planning. The absence of increased major morbidity supports its technical feasibility, while differences in oncologic outcomes emphasize the importance of careful patient selection. Future studies should focus on improving preoperative stratification by integrating imaging findings, extent of vascular involvement, and response to neoadjuvant therapy, as well as on standardizing the reporting of venous invasion patterns.

Several limitations must be acknowledged. All included studies were observational, with an inherent risk of residual confounding despite the use of propensity score matching in some cohorts. In addition, variability in neoadjuvant treatment protocols, surgical techniques, and pathological assessment may have influenced the results. Inconsistent reporting of venous involvement limited more refined subgroup analyses, and the lack of individual patient data prevented adjustment for key biological variables such as tumor grade or response to systemic therapy.

Despite these limitations, this meta-analysis provides clinically meaningful evidence supporting both the safety and oncologic validity of venous resection during pancreatoduodenectomy. The results underscore the importance of prioritizing oncologic principles and individualized patient selection rather than avoiding vascular resection per se. Future investigations should aim to standardize reporting and integrate biological and anatomical factors to further refine treatment strategies and improve patient outcomes.

## 5. Conclusions

Venous resection during pancreatoduodenectomy represents an anatomically driven extension of oncologic surgery rather than an independent determinant of adverse outcomes. The preservation of overall survival and comparable postoperative morbidity observed in this analysis supports the safety of venous reconstruction in appropriately selected patients treated in high-volume centers. Conversely, shorter disease-free survival and higher R1 rates appear to reflect the underlying biological aggressiveness of tumors requiring venous resection, particularly in the setting of segmental reconstruction. These findings underscore the importance of shifting the focus from a binary classification of venous resection toward a biology-oriented framework integrating patterns of venous involvement, tumor characteristics, and multimodal treatment strategies.

## Figures and Tables

**Table 1:** Characteristics of the studies included in the qualitative and quantitative synthesis.

Author (Year)	Country	Design	N (Total)	Comparator	Vascular Procedure	Outcomes Reported
Murakami 2015	Japan	Multicenter retrospective	937	PD vs. PD + VR	PV/SMV	OS, morbidity, mortality
Turley 2012	USA	Retrospective	204	PD vs. PD + VR	PV/SMV	OS, patency
Beane 2016	USA	NSQIP, PSM	1395	PD vs. PD + VR	PV/SMV	Morbidity, mortality
Rosenberg 2012	USA	Retrospective	204	PD vs. PD + VR	PV/SMV	OS, complications
Sakamoto 2011	Japan	Retrospective	250	PD vs. PD + VR	PV/SMV	OS
Selvaggi 2014	Italy	Prospective	60	PD + VR vs. palliative	PV/SMV	OS
Groen 2022	Netherlands	Nationwide cohort	1311	No VR vs. TVR vs. SVR	PV/SMV	OS, morbidity
Serenari 2019	Italy	Prospective	99	TVR vs. SVR	PV/SMV	OS
Zettervall 2019	USA	NSQIP	3002	VR vs. no VR	PV/SMV	30-day morbidity and mortality

Abbreviations: PD, pancreatoduodenectomy; VR, venous resection; PV, portal vein; SMV, superior mesenteric vein; OS, overall survival. Note: “30-day morbidity” refers to postoperative complications occurring within 30 days after surgery, while “mortality” refers to death within 30 days. When available, complications were classified according to the Clavien–Dindo classification.

**Table 2:** Summary of pooled effect estimates for oncologic and perioperative outcomes.

Outcome	Comparison	Effect Estimate	Interpretation
Overall survival	PD + VR vs. PD	HR ≈ 1.0 (NS)	VR not independent predictor
Overall survival	SVR vs. TVR	HR 2.01 (1.27–3.15)	Segmental VR worsens OS
Major morbidity	PD + VR vs. PD	OR~1.1 (NS)	Comparable morbidity
30-day mortality	PD + VR vs. PD	OR~1.0 (NS)	No increased mortality
PV/SMV thrombosis	SVR vs. TVR	Higher in SVR	Technique-dependent risk

Abbreviations: HR, hazard ratio; OR, odds ratio; CI, confidence interval; OS, overall survival. Note: Pooled estimates were calculated using a random-effects model. Statistical significance was defined as *p* < 0.05.

**Table 7:** Risk of bias assessment using the ROBINS-I tool.

Study	Confounding	Selection of Participants	Classification of Interventions	Missing Data	Outcome Measurement	Selective Reporting	Overall Risk of Bias
**Murakami** **2015**	Moderate	Low	Low	Low	Low	Low	Moderate
**Sakamoto 2011**	Moderate	Low	Low	Low	Low	Low	Moderate
**Turley 2012**	Serious	Moderate	Low	Low	Low	Low	Serious
**Beane 2016**	Low	Low	Low	Low	Low	Low	Low
**Serenari 2019**	Moderate	Low	Low	Low	Low	Low	Moderate
**Groen 2022**	Moderate	Low	Low	Low	Low	Low	Moderate
**Zettervall 2019**	Low	Low	Low	Low	Low	Low	Low

## Data Availability

The data supporting the findings of this study, including template data extraction forms, extracted datasets, and analytic materials used for all analyses, are available from the corresponding author upon reasonable request.
